# Zeolitic imidazolate framework derived ZnCo_2_O_4_ hollow tubular nanofibers for long-life supercapacitors†

**DOI:** 10.1039/d0ra01844a

**Published:** 2020-04-06

**Authors:** Shihang Zhao, Xianbo Yu, Hongmei Chen, Kai Tao, Yaoping Hu, Lei Han

**Affiliations:** School of Materials Science & Chemical Engineering, Ningbo University Ningbo Zhejiang 315211 China hanlei@nbu.edu.cn; Key Laboratory of Photoelectric Materials and Devices of Zhejiang Province, Ningbo University Ningbo Zhejiang 315211 China

## Abstract

Uniform one-dimensional metal oxide hollow tubular nanofibers (HTNs) have been controllably prepared using a calcination strategy using electrospun polymer nanofibers as soft templates and zeolitic imidazolate framework nanoparticles as precursors. Utilizing the general synthesis method, the ZnO HTNs, Co_3_O_4_ HTNs and ZnCo_2_O_4_ HTNs have been successfully prepared. The optimal ZnCo_2_O_4_ HTNs, as a representative substance applied in supercapacitors as the positive electrode, delivers a high specific capacity of 181 C g^−1^ at a current density of 0.5 A g^−1^, an excellent rate performance of 75.14% and a superior capacity retention of 97.42% after 10 000 cycles. Furthermore, an asymmetric supercapacitor assembled from ZnCo_2_O_4_ HTNs and active carbon also shows a stable and ultrahigh cycling stability with 95.38% of its original capacity after 20 000 cycle tests.

## Abstract 2

Uniform metal oxides hollow tubular nanofibers have been controllably prepared by calcination strategy using electrospun polymer nanofibers as soft templates and zeolitic imidazolate framework nanoparticles as precursors for long-life supercapacitor.

## Graphical abstract

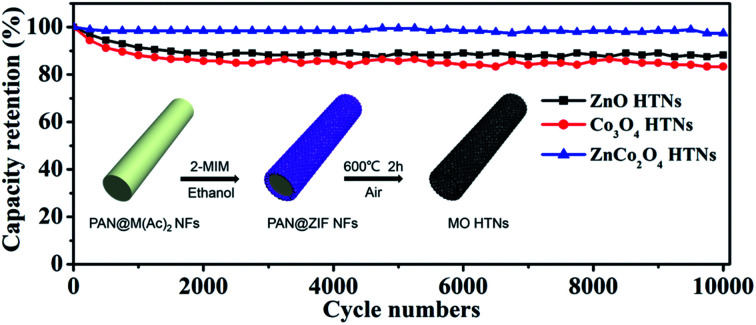

## Introduction

Since the overuse of the traditional fossil fuels like petroleum and coal, environmental issues have got more and more attention. Therefore, the study of environment friendly energy sources and storage devices has become a hot topic in the present society.^1–4^ Supercapacitors (SCs) have been considered as one of the candidates for next-generation energy storage devices and have been widely used in many fields.^5–8^ As is known, the electrode material is a crucial link in the whole SC device.^9,10^ Recently, transition metal oxides are favored by many researchers due to their high specific capacitance and energy density.^11,12^ For instance, an environment friendly material, ZnO, has received much attention because of its high conductivity and chemical stability.^13^ Co_3_O_4_ has an attractive application prospect in the field of energy storage due to ultra-high theoretical capacitance (3560 F g^−1^), low cost and various valence states of cobalt.^14,15^ Compared with single metal oxides,^16^ bimetallic oxides (such as ZnCo_2_O_4_) show more superior electrochemical performance as they deliver a higher electroconductivity on account of the comparatively low activation energy or electronic transport between metal ions, and they could provide multistep redox reaction.^17^ Despite all that, the large voluminal swell at cycling processes vastly hinders the practical application of transition metal oxides electrodes.

Hollow tubular structure materials have been studied extensively as a class of the structural electrodes materials in recent times.^18–20^ Especially, one-dimensional (1D) hollow tubular nanofibers (HTNs) structures contain merits including high surface area, low density and functional thin shell endowing them adequate potential to be applied in energy storage field.^21,22^ Nevertheless, the synthesis process of hollow tubular structures usually need hard templates or relatively complicated synthesis steps, which usually creates additional consumption.^23–26^ Therefore, it is highly desirable to explore facile and controllable synthesis strategy to form electrode materials with outstanding cycle performance. It is worth to note that electrospinning technique is a famous strategy for manufacturing nano-scale fibers with the uniform size, large surface area and highly controllability, and is applied to many fields such as lithium ion battery, solar cells and SCs.^27–30^ Some researchers have prepared 1D composite nanofibers by mixed the polymer solutions and electrochemical active materials, others have used the electrospun carbon nanofibers as a hard template to make structural fibers.^23,29^

Herein, we report a simple and effective method to prepare metal oxides HTNs (ZnO, Co_3_O_4_ and ZnCo_2_O_4_). By combining electrospinning technology and calcination process, the metal oxides HTNs are obtained by using polymer nanofibers as the soft template and zeolitic imidazolate framework (ZIF) as the precursor and metal source. Such uniform nanofibers can provide large specific surface areas and a shorter ions transport path. Furthermore, the excellent hollow tubular structure not only provides sufficient active sites for electrochemical reaction, but also effectively resists changes in the volume of the material during cycling process. For example, the ZnCo_2_O_4_ HTNs display a high specific capacity of 181 C g^−1^ and an outstanding cycling performance of 97.42% after 10 000 cycles as the positive electrode for SCs. Moreover, an asymmetric supercapacitor assembled by ZnCo_2_O_4_ HTNs and active carbon (AC) also shows a desirable electrochemical performance (ultrahigh cycling stability with 95.38% of original capacity after 20 000 cycle tests).

## Experimental

### Preparation of PAN@Zn(Ac)_2_/Co(Ac)_2_ composite nanofibers

The electrospinning solution is prepared by dissolving 0.45 g of polyacrylonitrile (PAN), 2 mmol of zinc acetate dihydrate (Zn(Ac)_2_·2H_2_O) and 4 mmol of cobalt acetate tetrahydrate (Co(Ac)_2_·4H_2_O) in 5 ml of dimethylformamide (DMF) with stirring for 12 h. The high voltage, flow rate, and distance between spinneret-to-collector are set as 20 kV, 0.8 ml min^−1^ and 15 cm, respectively.

### Preparation of PAN@ZnCo-ZIF core–shell nanofibers

In a typical synthesis, 2.5 mmol of 2-methylimidazole (2-MIM) is dissolved into 60 ml of ethanol, followed by the addition of 10 mg of the PAN@Zn(Ac)_2_/Co(Ac)_2_ composite nanofibers into the solution. Then, the mixture is kept at room temperature for 24 h, the obtained core–shell PAN@ZnCo-ZIF composite nanofibers are washed by ethanol for several times.

### Preparation of ZnCo_2_O_4_ hollow tubular nanofibers

The above PAN@ZnCo-ZIF core–shell nanofibers are thermal annealed in air at 600 °C for 2 h with a heating rate of 2 °C min^−1^ to obtain the ZnCo_2_O_4_ hollow tubular nanofibers. For comparision, the ZnO HTNs and Co_3_O_4_ HTNs are prepared in a similar process but only with the addition of Zn^2+^ or Co^2+^ ions.

### Materials characterization

The morphology and composition of the materials are characterized by scan electron microscopy (SEM, Hitachi S-4800), transmission electron microscopy (TEM, JEM-2100F) equipped with energy-dispersive X-ray spectroscope (EDS) detectors and high-resolution TEM (HRTEM). X-ray diffraction (XRD, Bruker D2 phaser) with Cu-Kα radiation measurements are carried out to examine the crystal phase of the samples. X-ray photoelectron spectrometer (XPS, PHI5600) is used to obtain the photoelectrically spectroscopy. Thermogravimetric analysis (TGA, Pyris Diamond) is carried out to verify the temperature of calcination. Nitrogen adsorption–desorption measurements are implemented on a Quadrachrome adsorption instrument (Autosorb-iQ3; Quantachrome, America) at 77 K. The pore-size distribution is calculated based on the Barrentt–Joyner–Halenda (BIH) method.

### Electrochemical measurements

The working electrodes (ZnCo_2_O_4_ HTNs and active carbon) are prepared by mixing the active materials (80%), acetylene black (10%), polyvinylidene fluoride (PVDF, 10%) and 1 ml ethanol with a stirring process of 10 h to form a slurry. Then spreading the slurry onto Ni foam and the electrodes are dried at 80 °C for 12 h under vacuum. The loading mass of ZnCo_2_O_4_ HTNs and active carbon is about 2.0 mg cm^−2^.

Electrochemical measurements are executed in a three-electrode system with a Pt foil as the auxiliary electrode and a saturated calomel electrode (SEC) as the reference electrode in an aqueous KOH electrolyte (2.0 M) by means of an electrochemical workstation (CHI 660D) at room temperature.

The specific capacity (*C*, C g^−1^) is calculated based on the following formula (1):^31^1
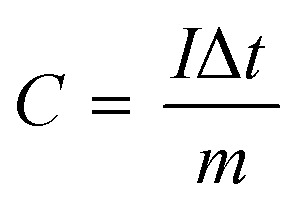
where *I* (A), Δ*t* (s) and *m* (g) represent the discharge current, discharge time and the mass of the active materials, respectively.

The asymmetric supercapacitor (ASC) device is assembled by using ZnCo_2_O_4_ HTNs as positive electrode and AC as negative electrode. The mass ratio of the positive and negative electrodes should be calculated by the following eqn (2) to achieve the charges balance of the two electrodes:^31^2
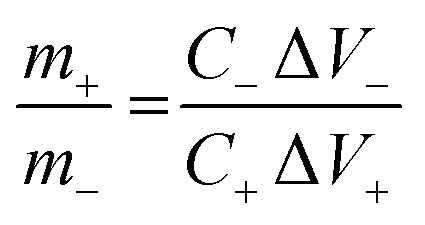
where Δ*V*_+_ (V) and Δ*V*_−_ represent the potential window of the positive and negative electrodes.

The energy density (*E*, W h kg^−1^) and power density (*P*, W kg^−1^) of the ASC device are calculated using the following eqn (3) and (4):^32^3
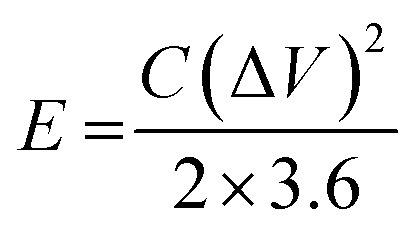
4
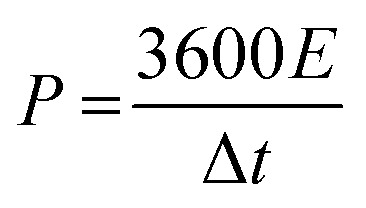
where Δ*V* (V) and Δ*t* (s) represent the potential window and the discharge time of the ASC device, respectively.

## Results and discussion

**Scheme 1:**
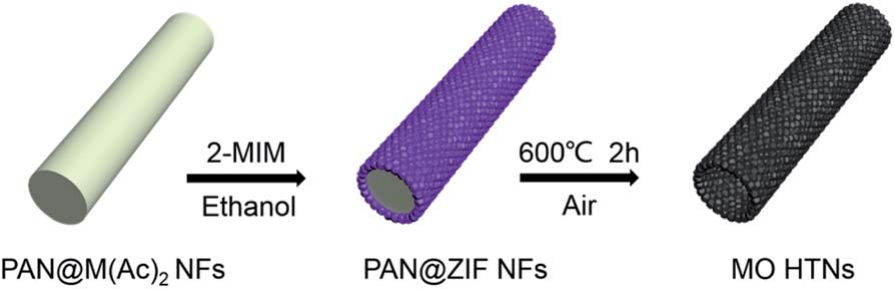
Schematic illustration of the synthesis of metal oxides HTNs.

The preparation method of metal oxides HTNs is shown in Scheme 1. Firstly, the electrospinning technology is utilized to prepare the PAN@M(Ac)_2_ composite nanofibers with uniform morphology and flexible mechanical character. Secondly, the as prepared PAN@M(Ac)_2_ composite nanofibers can be transformed into PAN@ZIF core–shell nanofibers by immersed in ethanol solution containing 2-MIM ligand. Finally, after a calcination process under the air, the core layer of PAN is burned off, and at the same time, the shell layer of ZIF is also oxidized to metal oxides, the HTNs framework constructed by metal oxides nanoparticles derived from ZIF materials come to being. By executing this synthesis strategy, we have successfully prepared the ZnO HTNs, Co_3_O_4_ HTNs and ZnCo_2_O_4_ HTNs, which means the current strategy is general, simple and effective, provides a guiding method for preparation of metal oxides hollow tubular nanofibers structure.

**Fig. 1:**
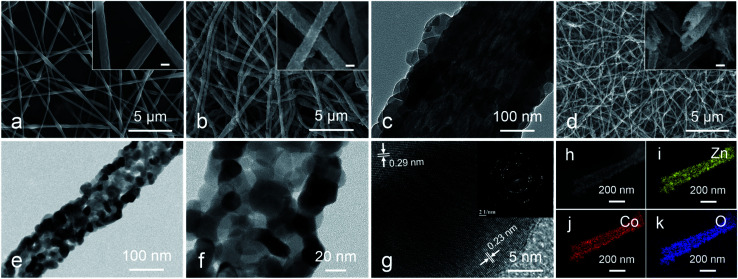
(a) SEM images of PAN@Zn(Ac)_2_/Co(Ac)_2_ composite nanofibers; (b) SEM and (c) TEM images of PAN@ZnCo-ZIF core–shell nanofibers; (d) SEM and (e and f) TEM images of ZnCo_2_O_4_ HTNs; (g) HRTEM image and SAED pattern (inset) of ZnCo_2_O_4_ HTNs; (h–k) elemental mapping images of ZnCo_2_O_4_ HTNs. Scale bars in inset of (a, b and d) are 200 nm.

The morphological features of three samples are characterized by SEM images, ZnCo_2_O_4_ HTNs are described as a typical sample. The smooth and flexible PAN@Zn(Ac)_2_/Co(Ac)_2_ composite nanofibers with a diameter of 200 ± 50 nm (Fig. 1a), can be synthesized in large quantity by electrospinning. After a coordination reaction between 2-MIM and metal ions (Zn^2+^, Co^2+^), ZIF nanoparticles grow on the surface of composite nanofibers uniformly, forming the PAN@ZnCo-ZIF core–shell nanofibers (Fig. 1b). This result can be verified by TEM image with a clear core–shell structure (Fig. 1c) and XRD pattern with a classical diffraction peaks of ZnCo-ZIF (Fig. S1a†).^33^ Through a calcination treatment in air, the 1D fiber-like morphology is well preserved, at the same time the ZnCo_2_O_4_ HTNs (Fig. 1d) structure is generated. The temperature of calcination is determined by TGA in air (Fig. S2a†). Before 200 °C, there is a slight weight loss, which is attributed to the evaporation of adsorption water or ethanol in the PAN@ZnCo-ZIF nanofibers, the second and third weight loss step is occurred around 270 °C and 350 °C, that because of the transform of ZnCo-ZIF into ZnCo_2_O_4_ and the decomposition of PAN nanofibers. After 400 °C, there is no evident weight loss. In order to ensure the PAN nanofibers are completely calcined clean, the 600 °C is selected at the suitable calcination temperature.^19,33,34^ The TEM images of the final product obviously reveal the successful preparation of the hollow tubular nanofibers structure and the thickness of the shell about 30 nm (Fig. 1e and f). The XRD analysis turns out that this final product is the expected ZnCo_2_O_4_ sample (Fig. S1d†).^35^ Moreover, two clear lattice stripes 0.23 nm and 0.29 nm can be observed in high-resolution TEM (HRTEM) image (Fig. 1g), and it corresponds the (311) and (220) lattice plane of the ZnCo_2_O_4_, respectively.^36^ For the selected area electron diffraction (SAED) study, the distinct diffraction ring confirms the crystal nature of the product effectively.^35,36^ In addition, the elemental mapping test further proves the homogeneous distribution of Zn, Co and O elements in the whole fibers (Fig. 1h–k). The nitrogen adsorption–desorption measurement results indicate the presence of large number of mesopores with pore size distribution concentrated at 3.5 nm and the high specific surface area of 34.191 m^2^ g (Fig. S3†). The mesoporous structure will expose the active sites inside the material, increase the contact area with the electrolyte and accelerate the ions diffusion kinetics.

**Fig. 2:**
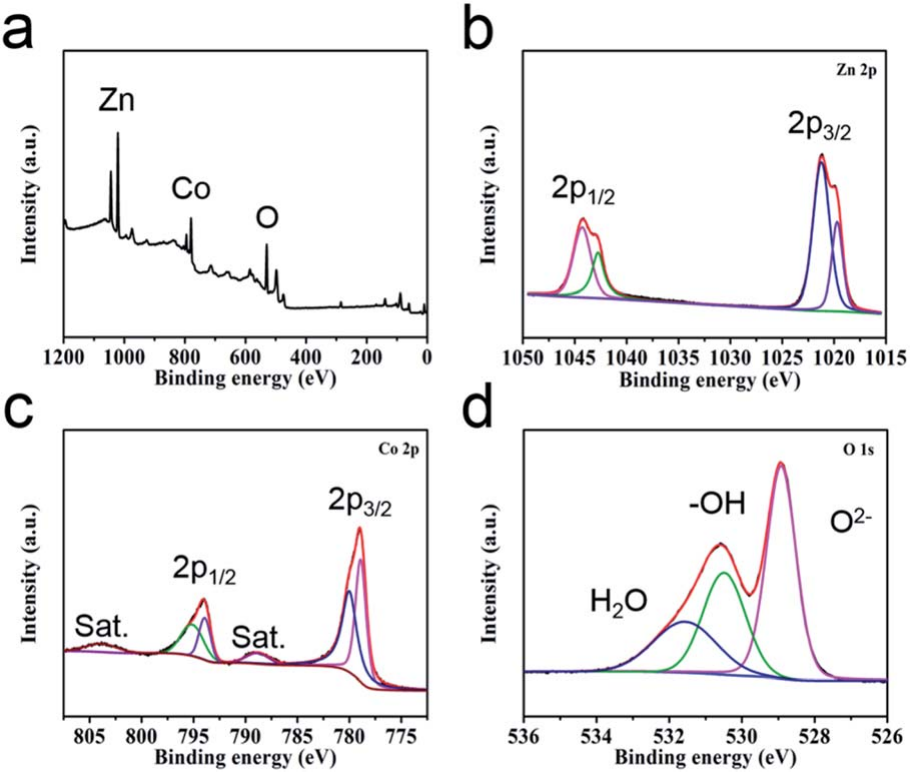
(a) XPS survey spectra of ZnCo_2_O_4_ HTNs and the high-resolution spectra of (b) Zn 2p, (c) Co 2p and (d) O 1s.

The valence states of the elements in the sample surfaces are revealed by XPS analysis. The survey image suggests the co-existence of Zn, Co and O elements (Fig. 2a), which is corresponding with the EDS study (Fig. S4†). The Zn 2p region spectra show two characteristic peaks, Zn 2p_1/2_ (1042.76 eV and 1044.3 eV) and Zn 2p_3/2_ (1019.7 eV and 1021.23 eV), suggesting the valence of Zn in the sample is +2 (Fig. 2b).^37^ For the high-resolution spectra of Co 2p (Fig. 2c), two spin doublets coupled with two satellite peaks could be observed. The bands located at 780.6 eV and 795.5 eV are attributed to Co^2+^, the peaks at 778.5 eV and 793 eV are ascribed to Co^3+^.^38,39^ The high-resolution spectra of O 1s can be divided into three peaks (Fig. 2d), metal–oxygen bond (O^2−^, 528.9 eV), hydroxyl group (–OH, 530.6 eV) and adsorption water (H_2_O, 531.8 eV).^40,41^ This study result further demonstrates the successful preparation of ZnCo_2_O_4_.

For comparison, the PAN@Zn(Ac)_2_ and PAN@Co(Ac)_2_ composite nanofibers are synthesized precisely by adjusting the kind of the adding metal element, after a solvent method treatment in room temperature, the PAN@ZIF-8 and PAN@ZIF-67 core–shell nanofibers are prepared, which is similar to the PAN@ZnCo-ZIF.^42,43^ Under the calcination process, the corresponding HTNs of ZnO and Co_3_O_4_ are synthesized resoundingly (Fig. S5 and 6†). Furthermore, a large number of basic test data (XRD, TGA and N_2_ adsorption–desorption measurement) strongly support the successful preparation of the ZnO HTNs and Co_3_O_4_ HTNs (Fig. S1b and c, S2b and c and S3†).^44,45^

**Fig. 3:**
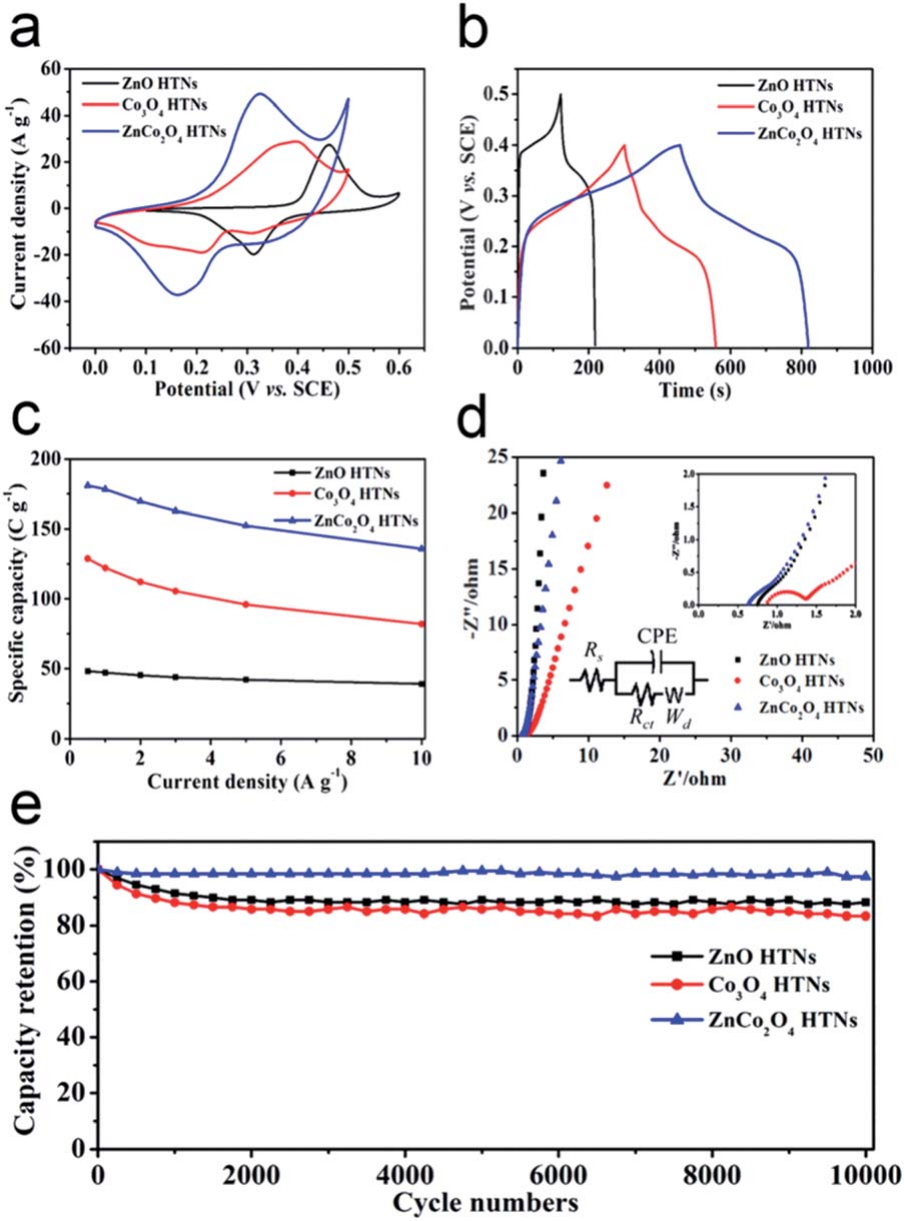
Electrochemical performance comparison of ZnO HTNs, Co_3_O_4_ HTNs and ZnCo_2_O_4_ HTNs: (a) CV curves at 50 mV s^−1^; (b) GCD curves at 0.5 A g^−1^; (c) specific capacity at different current densities; (d) EIS spectra in Nyquist plots; (e) cycling performance at current density of 5 A g^−1^.

The electrochemical behaviors of ZnO HTNs, Co_3_O_4_ HTNs and ZnCo_2_O_4_ HTNs, are studied by a three-electrode system in 2 M KOH electrolyte (Fig. S7–S9†). From the cyclic voltammetry (CV) curves of ZnCo_2_O_4_ HTNs (Fig. S7a†), the redox peak current increases with the increase of the scan rate (from 5 to 100 mV s^−1^), and at the same time, the height of peak density was shifted gradually. This phenomenon may be caused by the diffusion of the ions inside the electrode materials. The separated redox peak implies the reversible faradaic reactions occurred between the electrode materials and the electrolyte. The galvanostatic charge/discharge (GCD) tests are carried out at different current density (from 0.5 to 10 A g^−1^, Fig. S7b†). Compared with ZnO HTNs and Co_3_O_4_ HTNs, the sample of ZnCo_2_O_4_ HTNs exhibits the largest areas and the longest discharge times in CV and GCD measurements, respectively (Fig. 3a and b). This conclusion verifies that bimetallic oxide has a bigger advantage than single metal oxide in storage energy. As expected, ZnCo_2_O_4_ HTNs reached the highest specific capacity of 181 C g^−1^ compared with ZnO HTNs (48.2 C g^−1^) and Co_3_O_4_ HTNs (128.9 C g^−1^) at a current density of 0.5 A g^−1^ (Fig. 3c). In addition, when the current density is increased to 10 A g^−1^, the ZnCo_2_O_4_ HTNs, ZnO HTNs and Co_3_O_4_ HTNs also show an excellent capacity of 136 C g^−1^, 39 C g^−1^ and 82 C g^−1^, respectively, which means the three samples exhibit a high rate performance (75.14% of ZnCo_2_O_4_ HTNs, 80.91% of ZnO HTNs and 63.62% of Co_3_O_4_ HTNs). It is worth to note that for the ZnCo_2_O_4_ HTNs sample, the Zn atoms play a vital role in improving the conductivity and chemical stability of the electrode material.^44^ Relatively faradaic redox reactions are the following eqn (5) and (6):^46^

5 Co_2_O^−2^_4_ + 2H_2_O + OH^−^ ↔ 2CoOOH + e^−^

6 CoOOH + OH^−^ ↔ CoO_2_ + H_2_O + e^−^

By analyzing the CV curves at scan rate from 5 to 100 mV s^−1^, we can probe into the mechanisms of charge storage. The relationship between peak current (*i*) and scan rate (*v*) can be explored based on the power law (7):

7 *i* = *av*^*b*^

where *a* is constant, *b* is the power law exponent and it's also the slope of the linear plot of log(*i*) against log(*v*). In general, when *b* = 0.5, a diffusion-controlled contribution is happened, while *b* = 1 indicates a capacitive-controlled process. For ZnCo_2_O_4_ HTNs, the *b* values of cathodic is 0.718 (Fig. S10†), meaning the charge storage mechanism are both governed by diffusion-controlled and capacitive-controlled.^47^

Electrochemical impendence spectroscopy (EIS) tests are used to assess the charge transfer kinetics of electrodes in the frequency range from 0.01 Hz to 100 kHz (Fig. 3d). At low frequency range, the slope of the oblique line is correlated to Warburg impedance (*W*_d_), and the radius of a semicircle at high frequency range was relevant to the interface charge transfer resistance (*R*_ct_). Compared to the Co_3_O_4_ HTNs (*R*_ct_ = 0.1012 Ω) and ZnCo_2_O_4_ HTNs (*R*_ct_ = 0.01686 Ω) curve, the ZnO HTNs (*R*_ct_ = 0.01281 Ω) exhibit the smallest semicircle and biggest slope, suggesting that ZnO HTNs have the strongest charge transfer impetus at the interface of electrode/electrolyte. Moreover, for the EIS curves intersection with the *X*-axis corresponding to the equivalent series resistance (*R*_s_), ZnCo_2_O_4_ HTNs (0.9218 Ω) curve has a minimum value contrast with ZnO HTNs (1.112 Ω) and Co_3_O_4_ HTNs (1.508 Ω). This result further proofs the crucial impact of Zn atom for improving the material conduction. The cycling stability measurements are performed at 10 A g^−1^ for 10 000 GCD cycles. For the ZnCo_2_O_4_ HTNs electrode, it shows a particularly low capacity loss about 2.58% after cyclic testing (Fig. 3e). It is important to mention that ZnO HTNs (88.28%) and Co_3_O_4_ HTNs (83.33%) also have an outstanding cycling performance. This result is highly comparable in the respect of specific capacity, and this work has the longest cycling performance with relevant materials reported previously (Table S1†).

**Fig. 4:**
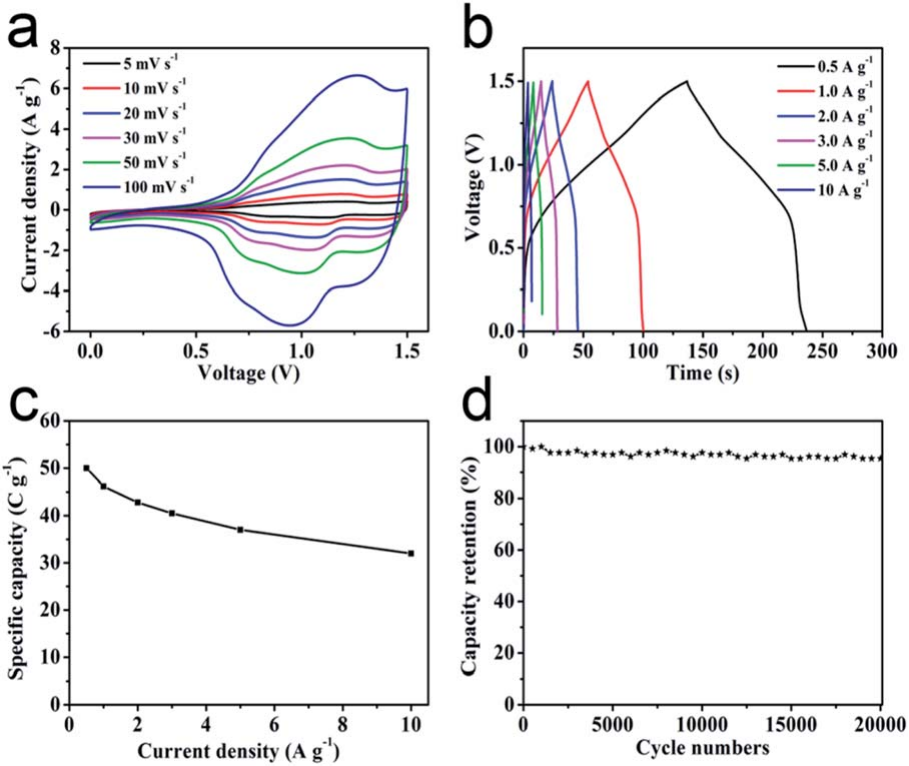
Electrochemical performance of the ZnCo_2_O_4_ HTNs//AC ASC: (a) CV curves at different scan rates; (b) GCD curves at different current densities; (c) specific capacity at different current densities; (d) cycling performance at the current density of 3 A g^−1^.

In order to investigate the practical application of metal oxides HTNs, we choose the ZnCo_2_O_4_ HTNs as a specimen to design an asymmetric supercapacitor (ASC), using the ZnCo_2_O_4_ HTNs and the active carbon (AC) as positive and negative electrode, respectively. For the electrochemical performance of AC electrode in three-electrode system (Fig. S11†), the approximate rectangular CV curves indicated the double-layer capacitance feature, the linearly symmetric GCD curves imply the good reversibility, and its specific capacitance is reached 182 F g^−1^ at a current density of 0.5 A g^−1^. By testing a series of different voltage window of CV and GCD curves at the same scan rate of 50 mV s^−1^ and the same current density of 1 A g^−1^ (Fig. S12a–c†), respectively, a slight polarization phenomenon was observed at 1.6 V, therefore, 1.5 V was preferred as the voltage window. The typical CV and GCD curves of ZnCo_2_O_4_ HTNs//AC ASC demonstrated an excellent reversible behavior (Fig. 4a and b). After calculation, the ASC delivers a high specific capacitance of 50 C g^−1^ at current density of 0.5 A g^−1^, this score reached to 32 C g^−1^ even increasing the current density to 10 A g^−1^ (Fig. 4c), which means the ASC owns a preeminent rate performance (64%). Moreover, benefitting from the stability of HTNs structure, the ASC device exhibits an ultra-high cycling performance, remained 95.38% of original capacity after 20 000 cycles at 3 A g^−1^ (Fig. 4d). For the Ragone plot, the ASC has a superior energy density of 10.42 W h kg^−1^ at power density of 375.12 W kg^−1^, and it still maintains 6.67 W h kg^−1^ at a high power density 7503.75 W kg^−1^ (Fig. S12d†), which present a high degree comparability with previously reported materials, such as porous ZnCo_2_O_4_ microspheres (6.22 W h kg^−1^ at 972.22 W kg^−1^),^48^ hollow ZnCo_2_O_4_ microspheres (12.62 W h kg^−1^ at 920.8 W kg^−1^),^49^ ZnCo_2_O_4_ ultra-thin curved sheets (10.2 W h kg^−1^ at 4250 W kg^−1^),^50^ ZnCo_2_O_4_/H:ZnO NRs (3.75677 W h kg^−1^ at 653.34 W kg^−1^).^51^

Such a prominent electrochemical property of transition metal oxides HTNs mainly put down to the excellent structural and compositional nature. The uniform nanofibers feature can provide a shorter diffusion path, the addition of zinc also greatly increase the chemical stability of electrode materials. Besides, the large specific surface area from the hollow structure can ensure the sufficient content between electrode and electrolyte, which resulting in a high rate capability. Specifically, the 1D tubular structure can store enough electrolytes, reduce the volume change of electrode materials during circulation, thus generating an outstanding cycle performance.

## Conclusion

In summary, we reported a simple and controllable calcination approach to prepare the transition metal oxides HTNs, utilizing electrospun polymer nanofibers as soft template as well as using ZIF nanoparticles as the metal sources. A representative substance, ZnCo_2_O_4_ HTNs deliver a desirable electrochemical performance, a high specific capacity of 181 C g^−1^ at the current density of 0.5 A g^−1^ with a capacity retention of 97.42% after 10 000 cycles when applied in SCs as the positive electrode. The ASC assembled from ZnCo_2_O_4_ HTNs and active carbon also shows a stable and ultrahigh cycling stability with 95.38% of original capacity after 20 000 cycle tests. Therefore, this synthetic strategy offers a direction for the study of hollow tubular nanofibers structure and long cycle life electrode materials.

## Conflicts of interest

There are no conflicts to declare.

## Supplementary Material

RA-010-D0RA01844A-s001
