# Supplementary material for: Zeolitic imidazolate framework derived ZnCo2O4 hollow tubular nanofibers for long-life supercapacitors
Source: RSC Adv. 2020 Apr 6;10(23):13922–8. doi: 10.1039/d0ra01844a (PMC9051560; doi:10.1039/d0ra01844a)
Supplement: RA-010-D0RA01844A-s001 [file RA-010-D0RA01844A-s001.pdf]

Electronic Supplementary Material (ESI) for RSC Advances.
This journal is © The Royal Society of Chemistry 2020

Supporting Information

Zeolitic imidazolate frameworks derived ZnCo2O4 hollow tubular nanofibers for long-life supercapacitors

Shihang Zhao,[a] Xianbo Yu,[a] Hongmei Chen,[a] Kai Tao,[a] Yaoping Hu[a] and Lei Han[a,b,*]

[a]School of Materials Science & Chemical Engineering, Ningbo University, Ningbo, Zhejiang 315211, China. E-mail: hanlei@nbu.edu.cn (L. Han).

[b]Key Laboratory of Photoelectric Materials and Devices of Zhejiang Province，Ningbo University, Ningbo, Zhejiang 315211, China.

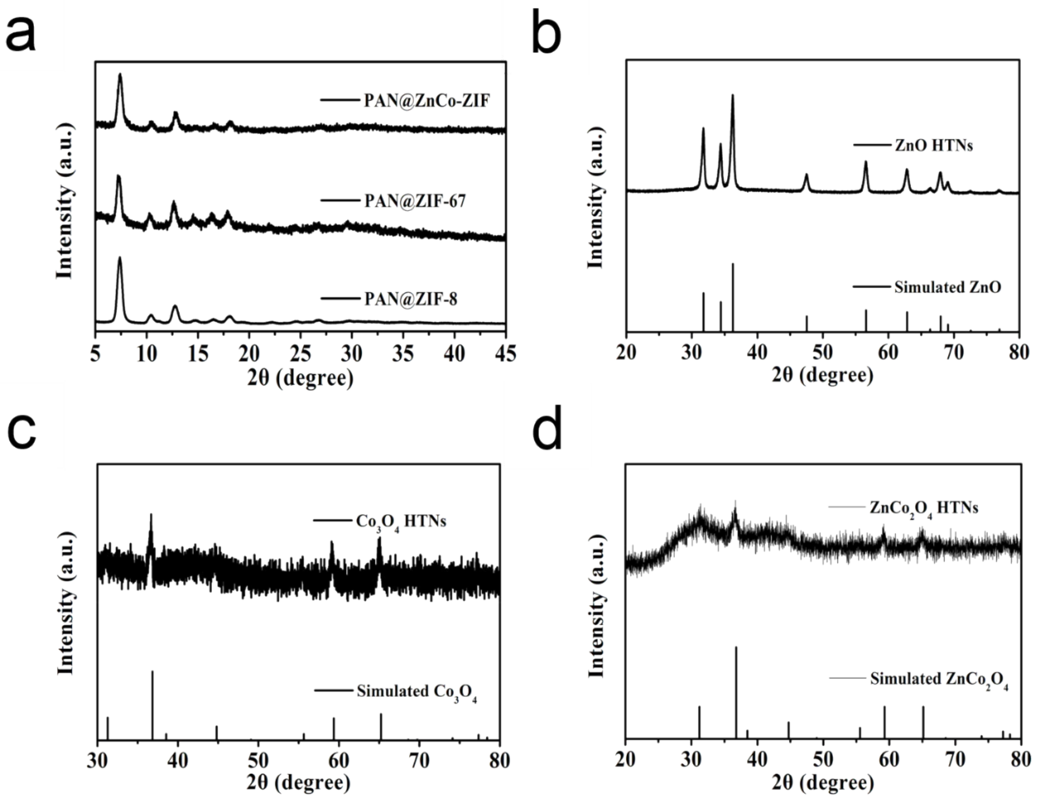

Fig S1. XRD patterns of (a) PAN@ZIF-8, PAN@ZIF-67 and PAN@ZnCo-ZIF, (b) ZnO HTNs, (c) Co_3O_4 HTNs, (d) $ZnCo_2O_4$ HTNs.

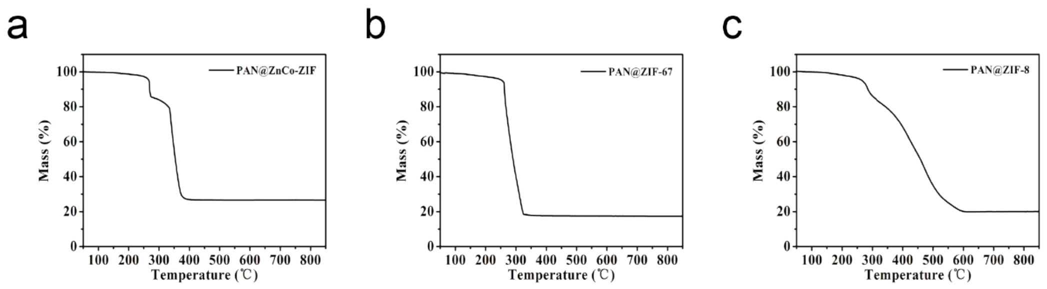

Fig S2. TGA curves of (a) PAN@ZnCo-ZIF, (b) PAN@ZIF-8 and (C) PAN@ZIF-67.

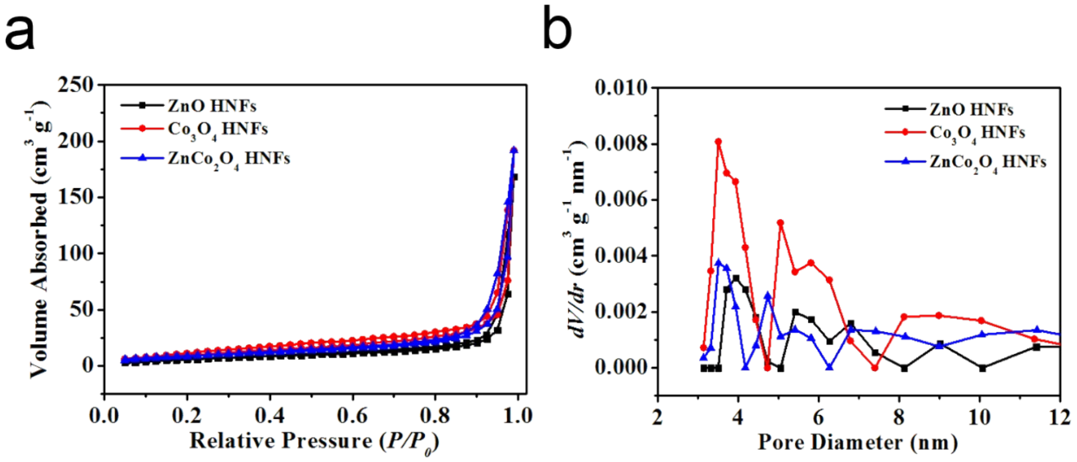

Fig S3. Nitrogen adsorption-desorption isotherms (a) and corresponding pore-size distribution curves (b) of $ZnCo_2O_4$ HTNs, ZnO HTNs and Co_3O_4 HTNs. (The pore size distribution of ZnO HTNs and Co_3O_4 HTNs are concentrated at 4 nm and 3.5 nm, and the specific surface area are 23.852 m^2 g and 38.979 m^2 g, respectively.)

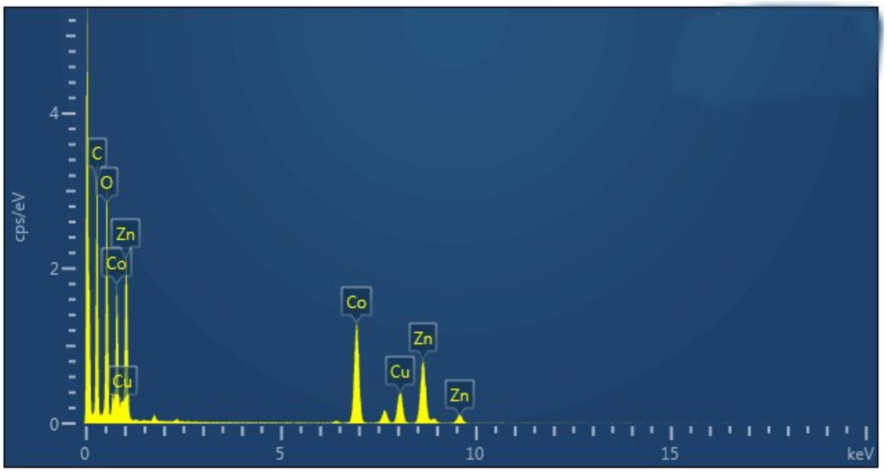

Fig S4. EDX image of $ZnCo_2O_4$ HTNs.

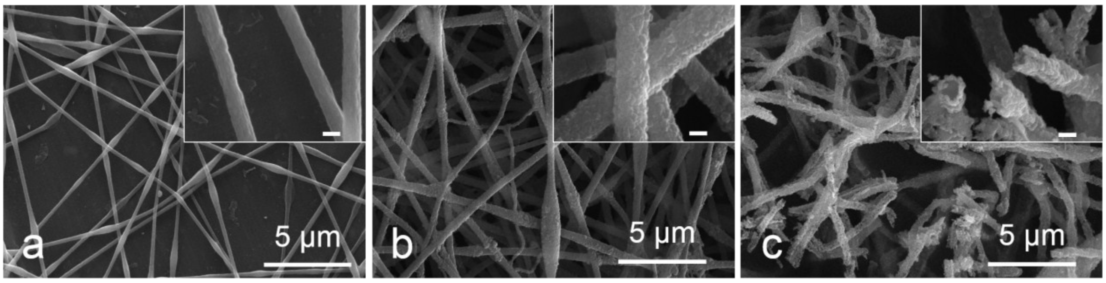

Fig S5. SEM images of (a) PAN@Zn$(Ac)_2$ composite nanofibers; (b) PAN@ZIF-8 core-shell nanofibers and (c) ZnO HTNs. Scale bars in inset are 200 nm.

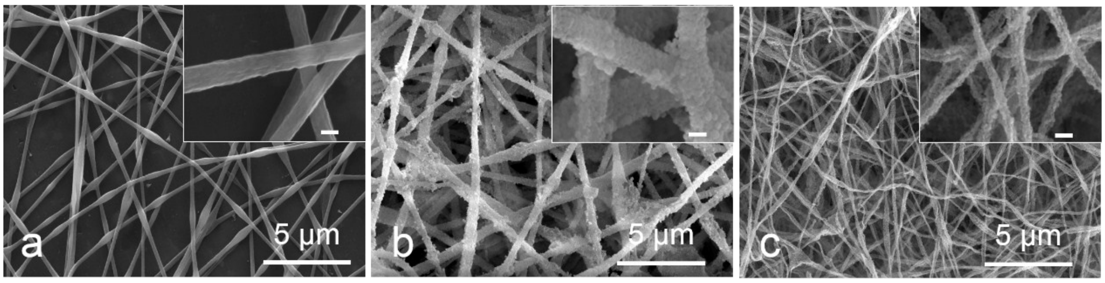

Fig S6. SEM images of (a) PAN@Co$(Ac)_2$ composite nanofibers; (b) PAN@ZIF-67 core-shell nanofibers and (c) Co_3O_4 HTNs. Scale bars in inset are 200 nm.

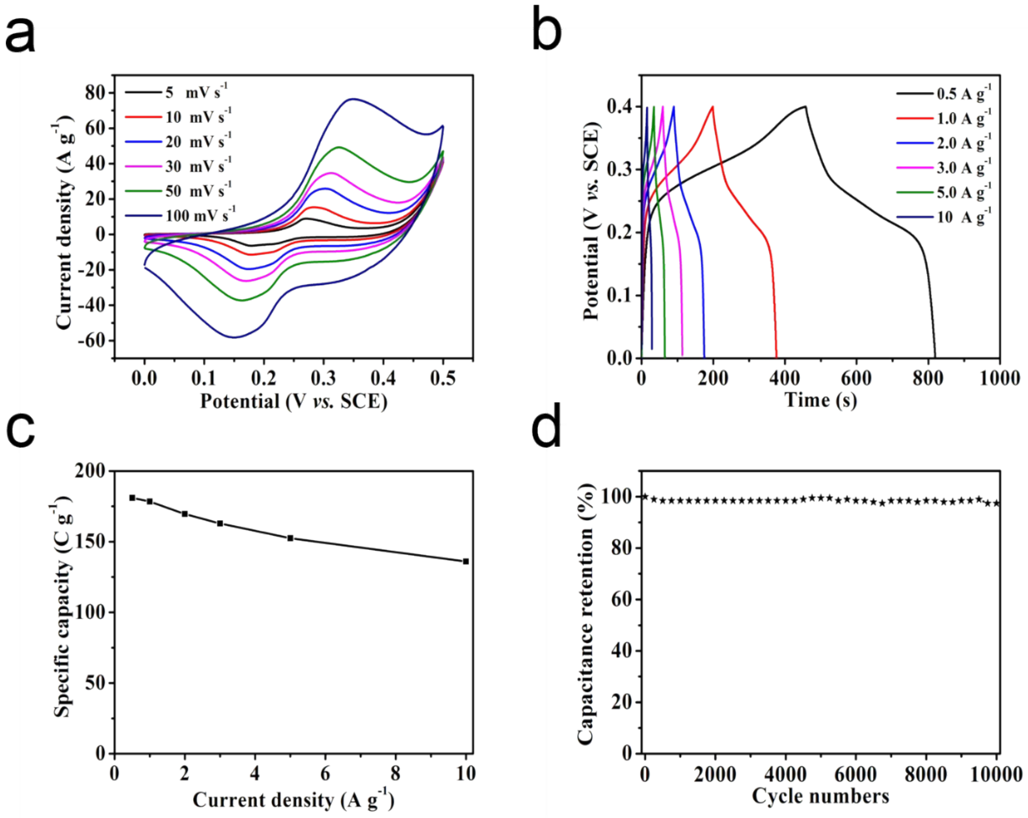

Fig S7. Electrochemical performance of $ZnCo_2O_4$ HTNs: (a) CV curves at different scan rates; (b) GCD curves at different current densities; (c) specific capacity at different current densities; (d) cycling performance at current density of 5 A g^{-1}.

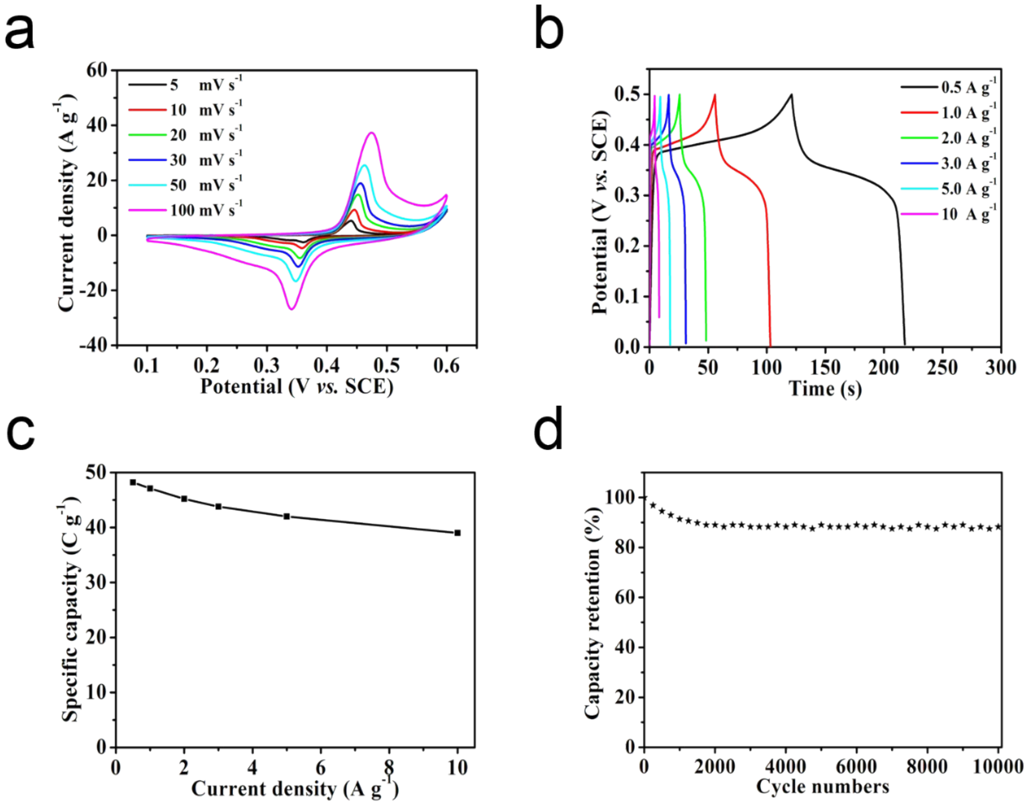

Fig S8. Electrochemical performance of ZnO HTNs: (a) CV curves at different scan rates; (b) GCD curves at different current densities; (c) specific capacity at different current densities; (d) cycling performance at current density of 5 A g^{-1}.

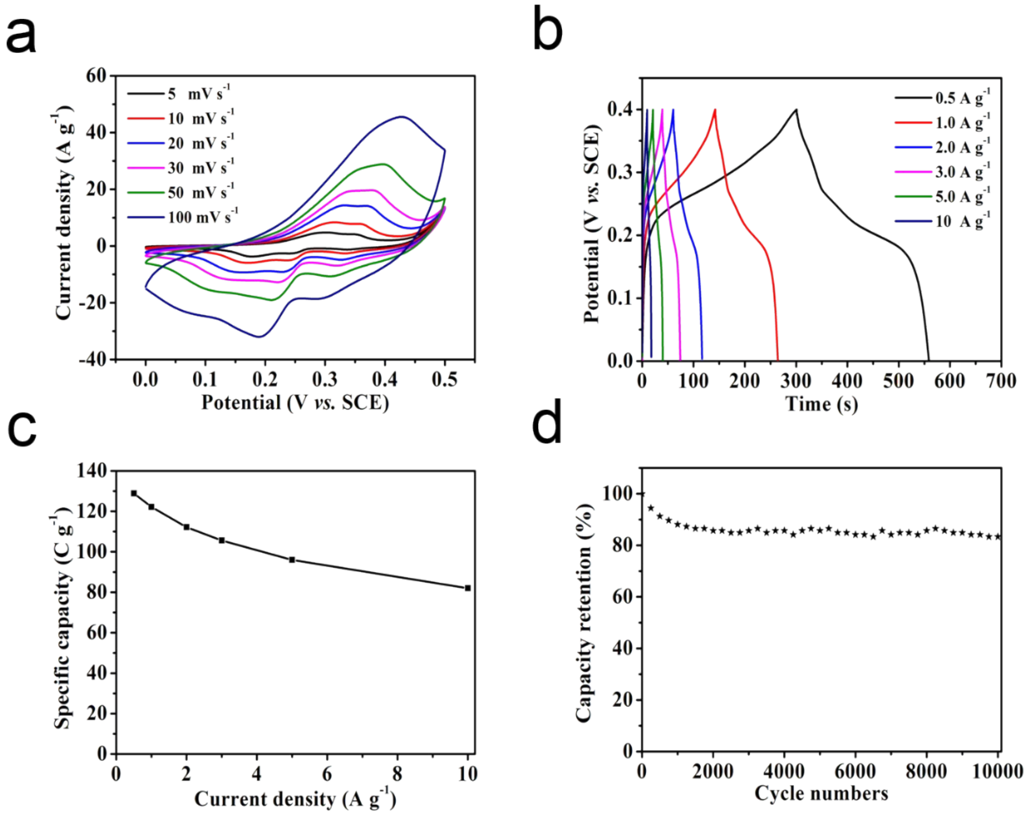

Fig S9. Electrochemical performance of Co_3O_4 HTNs: (a) CV curves at different scan rates; (b) GCD curves at different current densities; (c) specific capacity at different current densities; (d) cycling performance at current density of 5 A g^{-1}.

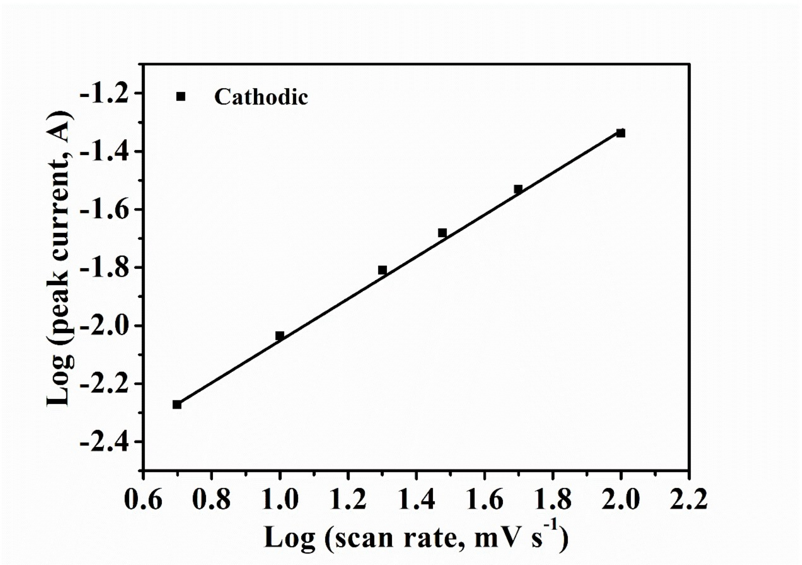

Fig S10. The plots of log(i) against log(v) of $ZnCo_2O_4$ HTNs.

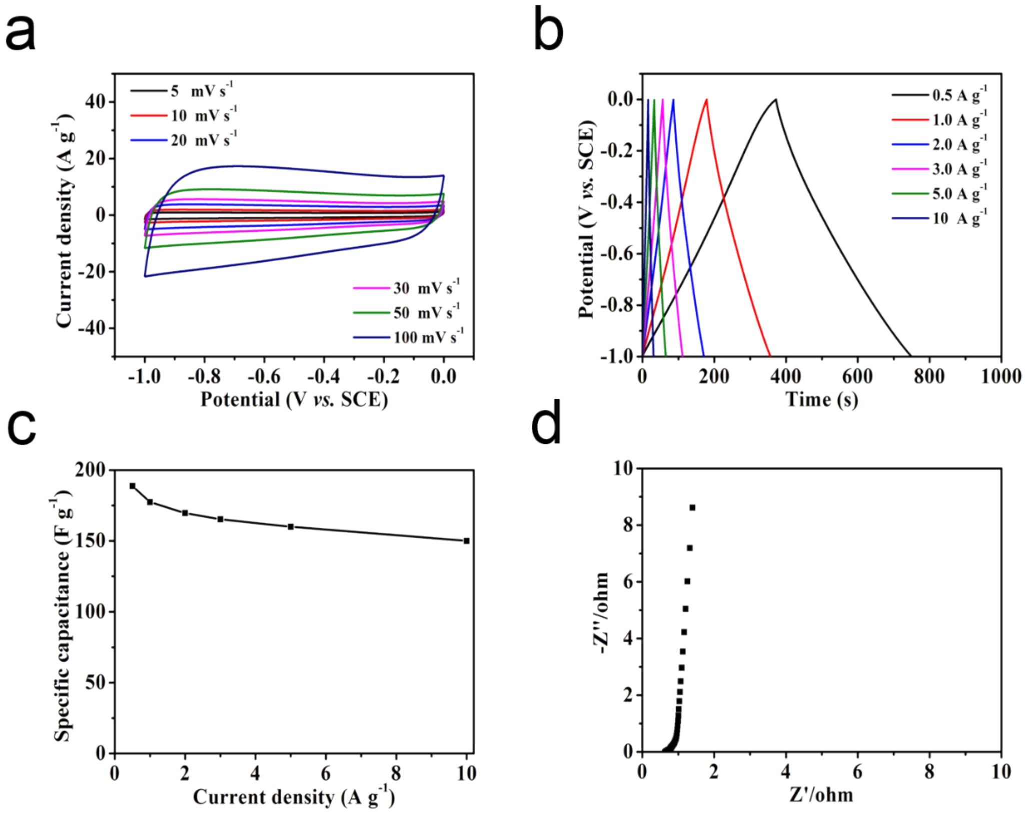

Fig S11. Electrochemical performance of AC: (a) CV curves at different scan rates; (b) GCD curves at different current densities; (c) specific capacitance at different current densities; (d) EIS spectra in Nyquist plots.

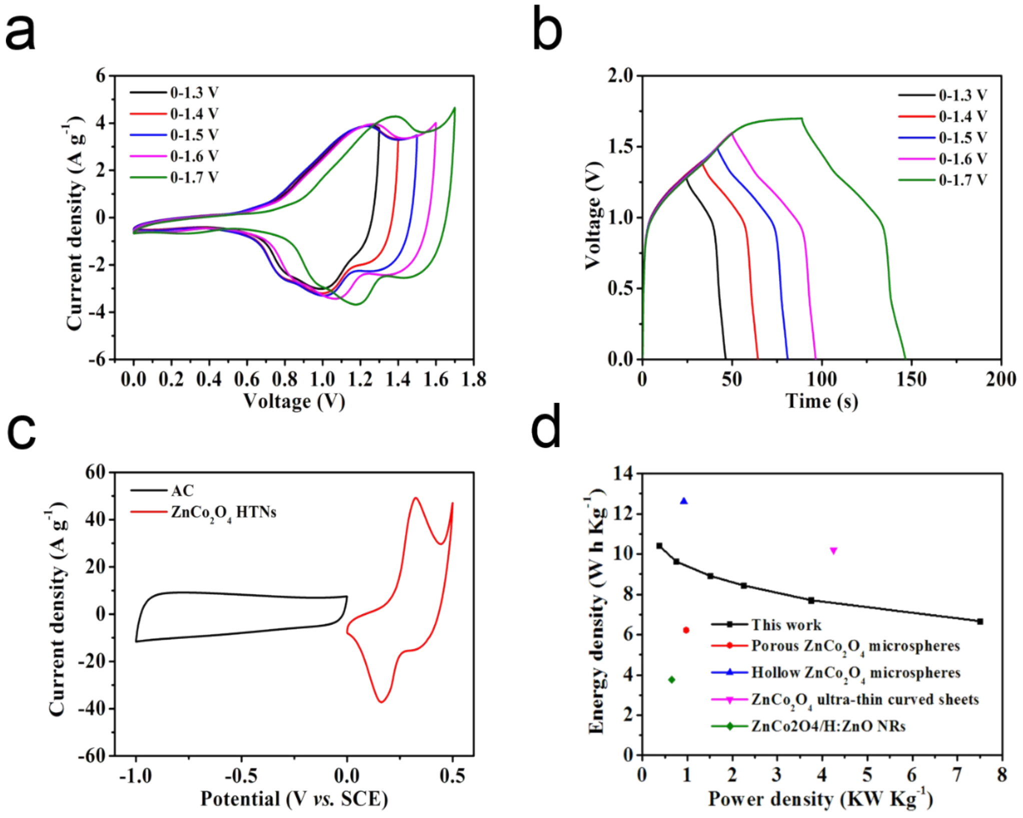

Fig S12. (a) CV curves with different voltage windows at the same scan rate of 50 mV s^{-1}; (b) GCD curves with different voltage windows at the same current density of 1 A g^{-1}; (c) CV curves of $ZnCo_2O_4$ HTNs and AC electrodes at the scan rate of 50 mV s^{-1}; (d) Ragone Plot of the $ZnCo_2O_4$ HTNs//AC ASC, in comparison with report data.

Tabel S1. Electrochemical performance comparison of $ZnCo_2O_4$ HTNs with related materials previously reported in supercapacitors.

Electrode	C (C g^{-1})	Current density	Cycle performance (cycle numbers)	Ref.
$ZnCo_2O_4$ nanoparticles	182.8	1 A g^{-1}	97.9% (1500)	S1
$ZnCo_2O_4$ microspheres	217	1 A g^{-1}	95.5% (2000)	S2
$ZnCo_2O_4$ nanoflakes	146.664	2 mA cm^{-1}	95.1% (2000)	S3
Peony-like $ZnCo_2O_4$	242	1 A g^{-1}	155.6% (3000)	S4
$ZnCo_2O_4$ microspheres	284	1 A g^{-1}	145% (2000)	S5
$ZnCo_2O_4$ nanowires	132	5 A g^{-1}		S6
$ZnCo_2O_4$ HTNs	181	0.5 A g^{-1}	97.42% (10000)	**This work**

References

S1 S. Chen, M. Xue, Y. Li, Y. Pan, L. Zhu, D. Zhang, Q. Fang and S. Qiu, *Inorg. Chem. Front.*, 2015, **2**, 177-183.

S2 Y. Gai, Y. Shang, L. Gong, L. Su, L. Hao, F. Dong and J. Li, *RSC Adv.*, 2017, **7**, 1038-1044.

S3 G. J. H. Lim, X. Liu, C. Guan and J. Wang, *Electrochim. Acta*, 2018, **291**, 177-187.

S4 Y. Shang, T. Xie, Y. Gai, L. Su, L. Gong, H. Lv and F. Dong, *Electrochim. Acta*, 2017, **253**, 281-290.

S5 Y. Shang, T. Xie, C. Ma, L. Su, Y. Gai, J. Liu and L. Gong, *Electrochim. Acta*, 2018, **286**, 103-113.

S6 Y. zhou, L. Chen, Y. Jiao, Z. Li and Y. Gao, *Electrochim. Acta*, 2019, **299**, 388-394.
